# Clinical Thermometry

**Published:** 1875-06-01

**Authors:** B. Kraus


### CLINICAL THERMOMETRY.

FROM THE "COMPENDIUM DER NEUEREN MEDICINISCHEN WISSENSCHAFTEN," BY DR. B. KRAUS.

*Condensed and translated by Dr. H. Gradle.*

### OSCILLATIONS OF PATHOLOGICAL TEMPERATURE.

AFTER having seen how little the physiological temperature is altered by individual peculiarities, or external influences, we shall find that on the contrary the variations in disease are considerable.

A temperature below 38° C. indicates that at the time no fever exists; the nearer, however, that this limit is approached the greater the probability that at another hour it has been exceeded, hence the necessity of repeated observation. While a temperature below 38.4° C. (101.1° F.) may denote merely a febrile tendency, anything beyond that point shows the existence of decided fever. The range of human temperature extends but in rare instances over more than 8° C. (14° F.). The highest animal heat observed by Wunderlich was 44.75° C. (112.5° F.) at the moment of death in a case of tetanus. Alvarenga considers a temperature of 42° C. as a rare occurrence, and not often has he seen the mercury rise to 43° C.; 44° C. has never come under his observation. The minimum temperature was found by Hardy in sclerema 22° C. (71.6° F.); Mignot saw 23° C. in the same disease. [A remark made at this place by the author that 44.5° C. (112° F.) is the maximum ever found in man during life, must be supplemented by a case of spinal injury, lately reported by Teale (*Brit. Med. Journal*, March 6th), in which the mercury reached in *different thermometers* the highest number of the scale, 122° F., and still recovery took place.]

The possible minimum of the temperature can, however, be determined but approximatively, since, according to Wunderlich, errors are most likely to occur at the lower degrees, and even the parts otherwise most reliable as indices do not indicate the correct blood temperature under these circumstances. In most cases the temperature does not fall below 35° C. in the well-closed axilla, and but rarely does it sink as low as 33° or 32° C. Though in some diseases, for instance cholera, a temperature of 26° C. and even less has been found on the surface, other observations render it probable that the vagina and rectum would have given a higher number.

The causes of morbid deviation of temperature are both external influences and constitutional conditions. A reduction can be occasioned through external means, either by increasing the loss of caloric, or by preventing the afflux of *warm* blood to the parts under observation, or, finally, by diminishing the production of heat. Severe degrees of external cold are the surest agent to abstract heat from the body, and when their duration is sufficiently prolonged they may even cause death. An external temperature, however, surpassing or even equalling the animal heat, possesses also a decidedly noxious influence, while it tends to raise the animal temperature. The application of external irritants seems to reduce rather than augment the internal heat. A mechanically induced, intense hyperæmia of a part raises its temperature, while a diminution of blood supply lowers it. Great loss of blood is followed, as a rule, by a lowering of the temperature; a spontaneous moderate hæmorrhage thus causes a reduction of the heat of febrile patients. Persistent starvation can also diminish the temperature considerably, while on the other hand the partaking of food may induce a moderate—and if the food be unsuitable, even a considerable—rise of temperature in disease and convalescence. A number of morbid accidents, as constipation, retention of urine, suppression of menses, etc., often occasion an augmentation of temperature, while diarrhœa (by purgatives), as well as other drains, vomiting (especially from the use of emetics), the influence of alcohol, some narcotics, and various other stronger or weaker poisons, can reduce the animal heat in a greater or less degree.

Amongst remedial agents we possess some capable of reducing a febrile temperature, for instance, digitalis, veratrin, quinine, calomel. A less marked influence is exerted by acids, potassic nitrate, and other salts, though in children, nervous women, and anæmic patients, their refrigerating effects are distinctly noticeable. Other agents, like coffee, musk, camphor, curare, etc., raise the temperature, both in health and disease.

In children and nervous or hysterical women, a marked unstability of animal heat is observable in disease. Not only are a greater rise and variation of temperature occasioned by unimportant affections, but also are the effects of extraneous influences exaggerated in them when compared with less excitable patients. Amongst male adults the nervous temperament and anæmia are also predisposing conditions for a similar impressibility. Advanced age, on the contrary, is accompanied by a greater stability of temperature, so that similar conditions result mostly in a temperature less by one-half degree C. than in younger individuals. Individual peculiarities also seem to exist, and experience has shown that while in some the repetition of certain influences diminishes the thermometric impressibility, in others it augments it.

Division of the cord, or severe injuries, are sometimes followed by a peripheral rise of temperature; in other cases nerve-injuries result in diminished warmth of certain parts, or of the entire body, according to their location. Muscular exertion often produces an increase of animal heat in feeble or sensitive individuals, which should always admonish us to look for other disturbances of health. As Wunderlich remarks, a disease, though not by itself modifying the temperature, may give rise to greater thermometric susceptibility to other influences. This same impressibility, though perhaps less marked, is also noticed in diseases which have by themselves altered the animal temperature. But the more typical the thermometric course of a disease, the less will be the influence of other accidents, while in atypical affections and slight disturbances of health the temperature depends considerably on the various extraneous conditions. In convalescence this impressibility is very considerable, the more so if the disease is still lurking in the organism. The nature of the influence is of some importance; if its direction is in conformity with the tendency of the disease, its effects are most manifest, and *vice versa*. Even the influence of diurnal oscillations is thus evident, an increase of temperature being most easily effected at noon and in the afternoon, while a depression of temperature is most decidedly brought about at night or early in the morning. Therefore the degrees observed at the time of the morning remission are of the highest importance.

Wunderlich has occasionally observed a temperature exceeding the highest degrees usually seen in fever, either not at all accompanied by other febrile symptoms, or, at any rate, by only those pertaining to a lower grade of fever; he applies to this the term *hyperpyrexia.*

### TEMPERATURE AS MEANS OF DIAGNOSIS AND PROGNOSIS.

A rise of temperature has always been considered as indicative of fever and its intensity, and since we have seen that in health the variations are minimal, we must look upon all deviations from the normal point as a symptom of disease: if over 37° C. of a febrile tendency proportional in intensity to the degrees exceeding this point; if under 36°, of an algid condition. An augmented heat is often the only symptom of a commencing or still lurking affection; thus the continued existence of an intermittent fever apparently cured is sometimes indicated by it alone. Yet no absolute references can be drawn from any given height of the mercury. While from a great augmentation or decline of temperature danger may be predicted, this is by no means always the case. The other circumstances of a case must therefore be taken into consideration.

In judging of the results of thermometric observation, the time of day must be considered, also the period of digestion—of so much influence in disease—as well as other accidental influences, especially removal of a patient, which may alter the temperature in either direction. A comparison of the existing temperature with the other symptoms offered must not be neglected, as by these means we may—if these are in marked contrast—detect simulation. If, however, the temperature is high, while no subjective symptoms exist, as is sometimes the case in infectious diseases, the onset of a severe affection may be predicted. Again, during convalescence from such conditions the opposite may occur, viz.: subjective illness co-existing with a normal or even *subnormal* temperature; in that case the state of the animal heat is the guide in our prognosis.

A contrast often exists between pulse and temperature. In the febrile state of adults 80 to 90 beats per minute usually correspond to a feebly febrile temperature, 90 to 108 beats to a moderate fever, 108 to 120 beats co-exist with a stronger grade, while a pulse over 120 indicates high pyrexia. In children, feeble and nervous individuals, the relation is not quite the same, the frequency of the pulse being mostly greater. Other differences also occur; thus exacerbations are sometimes first indicated by the pulse, while remissions may be primarily shown by a reduced temperature. Still less constant is the relation of temperature to frequency of respiration; in collapse an increase of the latter does often occur, though not invariably. In hyperpyrexia, also, could Wunderlich find no definite rules, the breathing being sometimes accelerated proportionately, sometimes retarded. Neither do brain-symptoms always correspond to the thermometric condition, as in adults delirium, etc., are usually concomitants only of a very high fever.

Abnormal deviations of temperature are occasionally noticed in apparent health; for instance, during menstruation, the puerperal state, lactation, dentition, the time of rapid growth, excessive fatigue, mental depression, etc., a rise is sometimes observed. The existence, therefore, of a normal temperature under these circumstances is a good evidence of perfect health, while a morbid increase calls for caution.

An augmented temperature, though not constantly followed by severe disease, calls in all cases for vigilance, and it is advisable to keep the patient in bed until the seguelæ are apparent. In the beginning of an acute febrile disorder, a diagnosis is rarely possible. If the temperature is normal, or but little higher, acute pneumonia, variola, and scarlatina can be excluded. If the normal degree is not at all or but little exceeded in the evening, typhus is not the disease. If, however, the mercury rises considerably at once, the range of possible affections includes the exanthemata, tonsilitis, pneumonia, pleuritis, intermittent fever, influenza, pyæmia, meningitis, typhoid, etc. In many cases the diagnosis is a matter of doubt in the earlier half of the first week. If, during this period, febrile symptoms are accompanied by a temperature normal in the evening, intermittent fever may be suspected, while typhus and typhoid, as well as the exanthemata (with the exception of measles, roseola, and varicella), can be excluded; catarrhal affections, measles, pleurisy, acute phthisis, granular meningitis, and acute rheumatism, are, however, possible. A sub-febrile or slightly febrile temperature is of similar significance; but, if occurring on the first or second morning, it does not speak against the onset of typhoid fever; this, however, is rendered unlikely by the occurrence of a high temperature within the first days, unless the disease can be shown to have commenced before it was suspected. If a diagnosis has been formed, the comparison of the existing temperature with the temperature incident to the disease at this time may be of service in determining the intensity of the affection.

In the second half of the first week the diagnosis may still be doubtful, the diseases possible being the initial fever of exanthemata, typhus, typhoid, relapsing and intermittent fever, pneumonia of slow onset, intense influenza, and capillary bronchitis, acute miliary tuberculosis, tubercular meningitis, cerebro-spinal meningitis, hepatitis, internal suppuration, osteomyelitis, acute syphilis. Of course a single observation is of little value in these cases. A temperature normal in the evening excludes the exanthemata, typhus and typhoid fever. Hyperpyrexia may indicate an intermittent or malignant infectious fever. If an eruption now occurs, it determines the nature of the disease.

After the formation of a diagnosis, continuous observation gives valuable information as to the progress, intensity, complications, danger, etc. Even a single thermometric examination may now be a useful hint to an examiner well conversant with the thermometric course of the affection; if comparatively high, it points to great severity; yet if, on the contrary, very low, it has less significance, as in most febrile disorders temporary refrigeration may occur. Typhus or typhoid fever can hardly be supposed, if the temperature between the third and tenth is not moderately febrile during day-time, highly febrile at evening (at least 39.6°), unless a reduction has been occasioned by free alvine discharges, hæmorrhage, or an advanced age of the individual. An unusually low temperature may give rise to a suspicion of internal hæmorrhage before the blood has made its way outward. A high morning temperature, near 40°, or an evening temperature of 41°, are grave symptoms in these affections. Of still more import is an excessive heat of the trunk, while the extremities become cold, in collapse. A temperature normal in the morning is not a positive sign of apyrexia. If, in measles, the temperature does not return to the normal after the appearance of the eruption, complications should be looked for. The same is true in scarlatina at a somewhat later period.

[*To be continued.*]

---

A SIMPLE METHOD OF REMOVING THE TEETH OF CHILDREN.—The operation consists in simply slipping a rubber ring over the tooth and forcing it under the edge of the gum. The patient is dismissed and told not to remove the appendage, which in a few days loosens the tooth and causes it to fall out. Grown children, who shrink from the shock and pain of the dental nippers, may also have their teeth removed by means of the rubber, which is a mild form of treatment.—*Druggists' Circular.*

